# Friends, Lovers or Nothing: Men and Women Differ in Their Perceptions of Sex Robots and Platonic Love Robots

**DOI:** 10.3389/fpsyg.2020.00355

**Published:** 2020-03-13

**Authors:** Morten Nordmo, Julie Øverbø Næss, Marte Folkestad Husøy, Mads Nordmo Arnestad

**Affiliations:** ^1^Department of Psychosocial Science, University of Bergen, Bergen, Norway; ^2^Department of Leadership and Organizational Behavior, BI Norwegian Business School, Campus Bergen, Norway

**Keywords:** robot, relationships, jealousy, gender differences, companionship, sex, artificial intelligence

## Abstract

Physical and emotional intimacy between humans and robots may become commonplace over the next decades, as technology improves at a rapid rate. This development provides new questions pertaining to how people perceive robots designed for different kinds of intimacy, both as companions and potentially as competitors. We performed a randomized experiment where participants read of either a robot that could only perform sexual acts, or only engage in non-sexual platonic love relationships. The results of the current study show that females have less positive views of robots, and especially of sex robots, compared to men. Contrary to the expectation rooted in evolutionary psychology, females expected to feel more jealousy if their partner got a sex robot, rather than a platonic love robot. The results further suggests that people project their own feelings about robots onto their partner, erroneously expecting their partner to react as they would to the thought of ones’ partner having a robot.

## Introduction

Advances in robot and artificial intelligence (AI) technology are moving at a rapid rate (Shoham et al., 2018). A number of scientists have predicted that robots will become an ordinary part of everyday social life, offering personalized service and companionship of different kinds (Schermerhorn et al., 2008; Flandorfer, 2012; de Graaf and Ben Allouch, 2013). Increasing sophistications of social AI such as Siri and Google Home invites the possibility of non-physical companionship between non-physical robots and humans. Companionship robots offer a promising avenue of innovation and research in fields such as child care, elderly care and certain branches of psychiatric care (Druin et al., 2000; Dautenhahn et al., 2006). One of the most fruitful promises of developing companionship robots is the alleviation of loneliness, which is especially prevalent among teenagers and the elderly (Victor and Yang, 2012) and has a detrimental effect on both physical and psychological wellbeing (Beutel et al., 2017).

As with many pioneering technologies before, applications of this technological advancement may be used to service both socio-emotional and sexual needs (Levy, 2007). Manufacturers intend to equip the more advanced sex robots with expanded options of movement and ability to converse appropriately with their owners. In the likely event that robots designed to satisfy human sexuality and emotion are commercialized, ethical, psychological, and social issues regarding human-robot interaction will emerge (Sullins, 2012; Richardson, 2016; Scheutz and Arnold, 2016; Danaher and McArthur, 2017; Frank and Nyholm, 2017). On a positive note, sex robots offer the promise of limiting or ending prostitution, sex-tourism and human trafficking associated with sex work (Yeoman and Mars, 2012. However, differences in psychological and moral perceptions of the use of sex robots may hamper market penetration. The field of research on perceptions of social robots is understandably limited, but the research holds some promise in both understanding how we view robot interaction. Contrasting findings on attitudes and psychological reactions to robot-human interaction may be informative to understand questions regarding general social topics as well. Based on earlier research (Nomura et al., 2006a), that showed a gender difference in attitudes toward robots, we posit that men and women will react differently to the prospect of robot human interaction. Scheutz and Arnold (2016) report the results from a survey of people’s attitudes toward sex robots. They found consistent evidence for a gender difference in how interested the respondents were in the prospect of sex robots, with men considering them more useful than women. While the results from this survey informed the basic premise of our study, we investigate the topic further by running a controlled survey experiment in which we vary the type of robot the participants read about. We therefore attempt to add to the literature by proposing the research question: How do men and women differ when evaluating the use of a platonic love robot or a sex-robot? In this research, we were interested in exploring gender differences in attitudes and predicted emotional reaction to two different kinds of social robots: (1) An AI sexual robot which can exclusively service physical sexual needs, and (2) an AI platonic love robot without a humanoid physical form which can form an intimate emotional bond with its owner, but are unable to engage in any sexual interactions in any form. Several factors motivated the direction of our exploration. Firstly, while a vast literature has amassed in psychology and sociology, describing gender differences in sexuality and social preferences, this literature has yet to be fully extended into the setting of human-robot interaction (Schermerhorn et al., 2008). Psychology offers a perspective on the perception and adoption of technology that is not always considered in technical circles. Understanding how users respond to robots and the reasons behind their responses will enable designers to create robots that fit well with the social, moral and relational climate they are targeting (Young et al., 2008). Understanding the role of gender differences in the perception of companionship robots and sex robots is not only necessary in order to tailor product development to different market segments, - it also offers a new and potentially fruitful avenue for understanding gender differences in basic needs and desires.

### Theory and Hypotheses

The overall aim of this exploratory study is to describe how men and women react to the possibility of robots designed exclusively for sex or love, and how they envision their partners’ reaction. Because these robots are not commercially available we designed the study to measure the predicted attitudes when imagining themselves and their partner interacting with it. Our study thus continues the exploration performed by Scheutz and Arnold (2016), in their survey of people’s attitudes toward sex-robots. In their survey, Scheutz and Arnold uncover a gender difference in how interested men and women are in sex robots, and how useful they are. However, the authors find evidence of gender convergence on the question of how interaction with a sex robot is to be classified and generally thought about. On this basis, Scheutz and Arnold suggest that larger views about robots, relationships and society, not just understandings of the robots themselves, should be a matter for more research. Our study represents an extension of this work, as it delves into the topic of how different types of robots with different capabilities are perceived and evaluated by men and women, using an experimental study design. We also add to the insights provided by Scheutz and Arnold (2016) by exploring peoples assumptions about their real or hypothetical partners reactions to the eventuality of them acquiring and using different kinds of robots.

Past research into gender differences in attitudes toward robots is limited. Nomura et al. (2006a) presented evidence suggested that in general, males were more positive toward interacting with the social robot; *Robovie* (Ishiguro et al., 2003). The present study represents a continuation of the findings provided by Nomura et al. (2006a), that showed a gender difference in attitudes toward robots. Based on their findings, we predicted that females would show greater general overall dislike to the thought of a robot, and find the thought of interacting with a humanoid robot less appealing. We therefore formulated our first hypothesis:

H1) Males will have more positive attitudes toward robots, compared to the attitudes held by females.

The experiment reported by Nomura and Kanda (2003) revolved around attitudes to a non-sexual, social robot. We wanted to explore gender differences toward robots designed to engage in different kinds of intimacy. In doing this, we wanted to bridge together insights from basic research on emotional intimacy and sexual preferences with novel questions arising from the advent of artificially intelligent robots. Previous research has documented predictable gender differences in preferences for emotional intimacy and sex (Buss et al., 1992; Petersen and Hyde, 2010). A key finding from this research is that men consistently have more frequent and more intense sexual desires than women do. This difference in sex drive is reflected in the reported prevalence of spontaneous thoughts about sex, frequency and variety of sexual fantasies, desired frequency of intercourse, desired number of partners, masturbation, pornography-use, attitudes toward casual sex, liking for various sexual practices, willingness to forego sex, initiating versus refusing sex, and making sacrifices for sex (Baumeister et al., 2001). In their 2016 survey, Scheutz and Arnold found evidence of a consistent difference between men and women in how useful and attractive the idea of a sex robot is. As the advent of sex robots has the potential to satisfice many sexual desires that otherwise would remain unfulfilled, it is reasonable to expect that males will continue to have more favorable attitudes toward sex robots.

Furthermore, we wanted to bridge the understanding of gender differences in preferences for platonic social and emotional intimacy to the prospect of platonic love robots. The idea of gender differences influencing the adoption of new technologies is not new. The history of technological product development already contain examples of how the adoption of products was affected by gender differences in social preferences. For instance, while the telephone was initially marketed as a professional tool reserved for male-dominated spheres, it was essentially appropriated by females to serve social ends (Fischer, 1988). Examples like these underline the importance in understanding gender differences when predicting the adoption of new products. Several strands of evidence from psychological research have suggested systematic gender differences in preferences for platonic emotional intimacy. Firstly, meta-analytic research on personality traits have found that females score higher than males on traits relating to a stronger social preference, such as extraversion, anxiety, trust, and, especially, tender-mindedness (i.e., nurturance) (Feingold, 1994). Females report having stronger and more rewarding friendships, especially with other females (Wright and Scanlon, 1991). Males score higher on self-compassion than females, which may provide some explanation for the gender difference in preference for social intimacy (Yarnell et al., 2015). Behavioral data also indicate gender differences in social needs and desires. Females self-disclose more than males, especially when talking to a person they have an established relationship with (Dindia and Allen, 1992). Females are also more inclined to seek emotional support from others as a way of coping with difficult emotions and general difficulties in life (Tamres et al., 2002; Nolen-Hoeksema, 2012). By contrast, men display a more avoidant adult attachment style, especially in intimate romantic attachment (Del Giudice, 2011). Research across multiple economic experiments demonstrate that females have a more other-regarding social preference (Croson and Gneezy, 2009). Meta-analytic findings from professional settings also provide support for the notion of gender differences in social preferences. Females have a more cooperative style of negotiating (Walters et al., 1998), a more democratic or participative style of leading (Eagly and Johnson, 1990), provide more psychosocial support as mentors (O’Brien et al., 2010), and endorse compromise more often as a conflict resolution strategy (Holt and DeVore, 2005). Research on attitudes toward seeking help in clinical settings can also provide direction to our second hypothesis, as meta-analytic suggests that females are more positive toward seeking professional help to alleviate psychological distress (Nam et al., 2010). Taken together, these findings provide plausible evidence for a slightly stronger preference for platonic social intimacy among females, compared to males, and a slightly stronger preference for pure sexual relationships among males, compared to females. On this basis, we formed our second hypothesis:

H2) Males will be more positive toward sex robots than platonic love robots, while females will be more positive toward platonic love robots than sex robots.

Both social robots and sex robots may appeal to males and females who live alone, or without a partner. Moreover, if these robots are to gain broad market appeal, they also need to be embraced by people living in committed relationships. Although men and women in committed relationships may not have the same social or sexual needs as individuals in relationships, they may still want to explore a social or sexual relationship with a robot. Loneliness and objective social isolation are often weakly correlated (Coyle and Dugan, 2012; Holt-Lunstad et al., 2015) and many people who have a partner report experiencing loneliness and sexual frustrations. Similarly, pornography use is widespread among heterosexual males in committed monogamous relationships, and many men who solicit prostitutes are married, which suggests that some sexual desires are not met by the sexual activities in the relationship (Sanders, 2013; Maas et al., 2018). By all accounts therefore, it is possible that both males and females in committed relationships may come to harbor a desire to include a sex robot or a social robot in their daily life as committed partners. Actual demand, however, will be very much contingent on how the partner feels about the presence of the robot. We therefore also explored how males and females would feel about their partner acquiring and using a social robot or sex robot. Psychological research has a rich tradition for exploring gender differences in jealousy, defined as negative feeling or suspicion that one’s partner is attracted to or involved with someone else (Buss et al., 1992). The general finding from evolutionary psychology suggests a slight difference between males and females in propensity to experience jealousy in different situations. Males tend to feel more jealousy when thinking about or experiencing their partners sexual infidelity, as compared to emotional/romantic but non-sexual infidelity. Females show the opposite pattern. On this basis we formed our third hypothesis:

H3) Males will expect to feel more jealous if their female partner gets a sex robot, while females will expect to feel more jealous if their male partner gets a platonic love robot.

Although several knowledgeable experts have claimed that artificially intelligent robots will be developed in the near future (Levy, 2007), and despite the popular appeal of fiction television series and movies that portray such a future, it can be difficult for research participants to envision and predict specific emotional reactions to these scenarios. It may also be that research participants are able to predict their general emotional valence (positive/negative) to the prospect of their partner having a robot, but that they disagree with labeling the negative emotion jealousy. In order to partially circumvent this validity threat, we also explored how participants felt in general about the prospect of their partner having a robot. We also explored how the participants theorized that their partner would react to them having a robot. This latter measure is presumably important for the market success of the robots; if one expect that one’s partner would hate the idea of a robot, then one would presumably never even entertain the topic and explore the accuracy of those expectations. Our theoretical predictions of general liking and disliking of one’s partner having a robot was rooted in the same evolutionary psychological account that formed the basis for the predicted gender differences in jealousy. As such, we expected males to dislike the idea of their female partner having a sex robot more than they would dislike her having a social robot. For female participants, we expected the opposite pattern. Our fourth hypothesis was thus:

H4) Males will be more negative to the prospect of their female partner getting a sex robot, while females will be more negative to the prospect of their male partner getting a platonic love robot.

Lastly, we wanted to explore differences in expectations about how their partner would feel if they decided to have a robot. This issue is of importance, as many people in committed relationships presumably will avoid purchasing a robot that they expect their partner will dislike them having. Their theories about their partners feelings will thus guide their behavior. When people theorize about the preferences of others, in settings where they don’t have good information to guide their theorizing, they tend to project their own feelings and goal states to the other person (Newman et al., 1997; Maner et al., 2005). Especially when particular emotions and goals are activated and made salient, people tend to over-perceive similar emotions and goals in others (Niedenthal et al., 2000; Kawada et al., 2004). As our participants presumably did not have accurate and updated information about how their partner would feel about them getting a robot, we expected that participants would theorize that their partners feelings about them having a robot would mirror their own. We thus postulated our fifth and final hypothesis:

H5) Males will expect that a partner would respond more negatively to him having a platonic love robot, while females will expect that a partner would respond more negatively to her having a sex robot.

## Materials and Methods

### Participants

We performed a vignette experiment with 163 female and 114 male participants. Recruitment of participants was accomplished by online distribution of the study. Mainly, we published the study on social media and distributed it by e-mail. The participants’ age varied from 17 to 70 years with a mean of 27.29 (*SD* = 9.8) years. The majority (68%) of the respondents were students. Most participants were heterosexual (90%), a few participants were homosexual (2%) and some did not identify as either sexuality (8%). Participation was voluntary and anonymous.

### Design

The experiment included two conditions to which the participants were randomly assigned; one in which they were exposed to a vignette about a futuristic sex robot, and in the other condition to a vignette about a love robot with advances social and emotional competencies, but without a humanoid physical form or ability to engage in sexual interaction. We purposefully described the robots as being either exclusively for sexual use, or exclusively for platonic love. The vignettes were presented with associated visual stimuli; a sexualized photo of an artificial looking man and a woman (sex robot) and a photo of ear plugs (platonic love robot). Extracts of the vignettes are available down below, while full versions and photos are presented in the Appendix.

#### Sex Robot

Imagine the year 2035. The world has seen great advances in artificial intelligence and robotics. One of the advances has led to the development of highly realistic sex robots, both in male and female form. The robots looks and feels just like humans (.). The artificial intelligence the robots are equipped with enables them to learn their owner’s sexual preferences through experience (.). User surveys show that the owners of this kind of sex robot are extremely satisfied (.). Even though the sex robots are equipped with a highly sophisticated artificial intelligence, there are some limitations to them. The robots can only have a sexual relationship with their owner. Attempts of non-sexual interactions will either be misunderstood, ignored or interpreted in a sexual way by the robot (.). The robots cannot form a meaningful romantic or friendly relation with a human.

#### Platonic Love Robot

Imagine the year 2035. The world has seen great advances in artificial intelligence and robotics. One of the advances has led to the development of highly realistic love robots, both in male and female form. The robots able to talk to their owners in a way that feels very human-like and realistic (.). The artificial intelligence the robots are equipped with enables them to get to know their owner through experience (.). User surveys show that the owners of this kind of love robot are extremely satisfied (.). Even though the love robots are equipped with a highly sophisticated artificial intelligence, there are some limitations to them. The robots have no physical body, it only exist in a small microphone and speaker (.). It can form a meaningful romantic and friendly relation to a human, but it cannot satisfy the owner in a sexual manner.

### Measurements

After reading about either the love robot or the sex robot, the participants were asked to think of a committed romantic relationship they have had at a previous time, are engaged in now or wish to have in the future. They were then asked to fill out a questionnaire regarding how they imagine themselves reacting if their partner owned and used a robot similar to the one they had read about, and how they think their partner would react if themselves interacted with such a robot on a regular basis. All items were recorded on a seven point scale from: (1) *Totally agree* to (7) *Totally disagree* as well as (4) *neither agree nor disagree*. All items were presented in Norwegian and were translated to English using a translation process in accordance with the recommendations made by Douglas and Craig (2007).

#### Robot Attitudes

Attitudes toward robots were measure with three items; *I hope this type of robot is developed in the future. I look forward to the development and launch of this type of robot*. *I feel we should not develop this type of robot* (reversed). Cronbach’s alfa for the measure was 0.94.

#### Jealousy

Robot jealousy was measured with the three items; *This kind of robot would evoke strong feelings of jealousy in me. I think I would feel jealous of this robot. I will not become jealous of this robot* (reversed). Cronbach’s alfa for the measure was 0.92.

#### Dislike of Partner’s Use

Dislike of a partner’s use was measured with three items; *I alone should take care of this kind of needs for my partner. I would like my partner to get rid of this robot. I do not mind my partner using this robot* (reversed). Cronbach’s alfa for the measure was 0.90

#### Predicted Partner’s Dislike of Own Use

How individuals predict their partner’s reaction to their own use of a robot was measures with three items: *My partner would not like it if I used this type of robot. My partner would want me to get rid of the robot. I think my partner would like me using this robot* (reversed). Cronbach’s alfa for the measure was 0.90.

#### Belief in Robot Technology

We also measured to what extent the participants in the experiment believe this kind of robot will be developed in the future, with three items; *I think we will see such robots developed in the future. Robots like these are going to be on the marked soon. We will never see this type of robot in production* (reversed). Cronbach’s alfa for the measure was 0.92.

#### Control Question

The participants also answered a control questions after being presented with the experimental stimulus, before the questionnaire. Participants were asked the control question (*The robot I read about can only engage in sexual relations*, (1) Correct, (2) Incorrect, (3) I do not remember). Wrong answers and admission of not remembering led to removal from the dataset.

#### Demographics

Lastly, the participants recorded age, gender, sexual orientation and student status (y/n). The survey was estimated to take 5-7 min to complete. The participants received no reward or compensation for participating.

### Statistical Analysis

Due to differences in number of male and female participants, we testes the assumption of equal variance between genders in preliminary analysis with Levene’s test and found no unequal variances in the four outcome variables. We analyzed the experiment data with full factorial regression analysis. Two models were tested for each of the four outcome variables: Belief in robot technology and age constitutes validation variables, while main effects and the interaction between gender and experimental condition investigate the research questions. We used marginal estimates to graphically plot the interaction effect. Differences between predicted estimated values are tested with *F*-tests. We also present descriptive information and pairwise correlations between the studies variables. Alfa was set to 0.05.

## Results

The high mean of belief in robot technology show that most of the participants believe that robots designed for intimacy are realistic, both in sex robot and platonic love robot format. This provides some support for the validity of the study. In line with past research on the topic, the general attitudes toward the robots were negative, regardless of the gender of the participants and type of robot. Attitudes toward the robot were positively correlated with a belief in robot technology and negatively correlated with dislike of their use, predicted level of partners dislike, as well as jealousy. We did not find any significant correlations between age and the robot attitudes and belief in robot technology. The descriptive statistics and pairwise correlations are all presented in Table 1.

**TABLE 1:** Descriptive Statistics and pairwise correlations.

**Variables**	**Mean**	**SD**	**(1)**	**(2)**	**(3)**	**(4)**	**(5)**	**(6)**	**(7)**	**(8)**
(1) Robot attitudes	10.19	4.73	1.00							
(2) Belief in robot technology	17.16	3.31	0.29**	1.00						
(3) Dislike partners use	17.56	3.66	−0.45**	–0.05	1.00					
(4) Predicted partners dislike	17.19	3.59	−0.28**	0.08	0.55**	1.00				
(5) Jealousy	14.80	4.81	−0.20**	0.05	0.58**	0.29**	1.00			
(6) Gender	1.63	0.48	−0.34**	−0.18**	0.20**	–0.08	0.13*	1.00		
(7) Experimental condition	1.50	0.50	–0.06	0.04	0.07	0.19**	0.08	–0.04	1.00	
(8) Age	27.29	9.80	0.05	–0.10	–0.08	–0.004	–0.08	−0.21**	0.04	1.00

*p > 0.05 **p > 0.01

### Hypothesis Testing

The results from the main effect and interaction effect models are presented in Table 2. Hypothesis 1 stated that males will have more positive attitudes toward robots, compared to the attitudes held by females. In support of this we found a significant negative main effect of gender on attitudes toward robots [*B* = −2.97, *p* < 0.01]. This finding demonstrates that males are more positive toward robots than females, regardless of experimental condition and type of robot they envision. In addition to attitude, the results also showed a negative main effect of gender on both dislike if their partner had a robot [*B* = 1.51, *p* < 0.01], and jealousy [*B* = 1.59, *p* < 0.05].

**TABLE 2:** Main and interaction effects of gender, experimental condition, with age and belief in robot technology as control variables.

	**Jealousy**		**Robot attitudes**		**Dislike partners use**		**Predicted partners dislike**	
	**Main effect**	**Interaction**	**Main effect**	**Interaction**	**Main effect**	**Interaction**	**Main effect**	**Interaction**
Intercept	9.50**(2.47)	12.71**(2.03)	10.54**(2.28)	5.38**(2.92)	14.88**(1.93)	17.77**(1.58)	15.06**(1.85)	16.72**(1.51)
Age	−0.01(0.03)	−0.01(0.03)	0.005 (0.02)	0.005 (0.17)	−0.01(0.02)	−0.01(0.02)	−0.008(0.02)	−0.007(0.02)
Belief in robot technology	0.11 (0.08)	0.11 (0.08)	0.33**(0.08)	0.33**(0.08)	−0.024(0.06)	−0.025(0.06)	0.06 (0.06)	0.062 (0.06)
Gender	1.59(0.62)*	0.58 (0.86)	−2.97**(0.57)	−1.09(0.57)	1.51**(0.48)	0.514 (0.67)	−0.49(0.46)	−1.66**(0.63)
Experimental condition	0.98 (0.57)	−0.28(0.95)	−0.92(0.53)	1.45 (0.54)	0.74 (0.45)	−0.511(0.73)	1.39**(0.43)	−0.085(0.70)
Gender*Condition		1.99 (1.19)		−3.79**(0.52)		1.98*(0.93)		2.35**(1.51)
Adjusted *R*^2^	0.02	0.03	0.16	0.20	0.04	0.05	0.03	0.05

Males and platonic love robots are reference groups for gender and experimental condition. SD in parenthesis. *N* = 163 women and 114 men. * *p* < 0.05; ** *p* < 0.01

**FIGURE 1:**
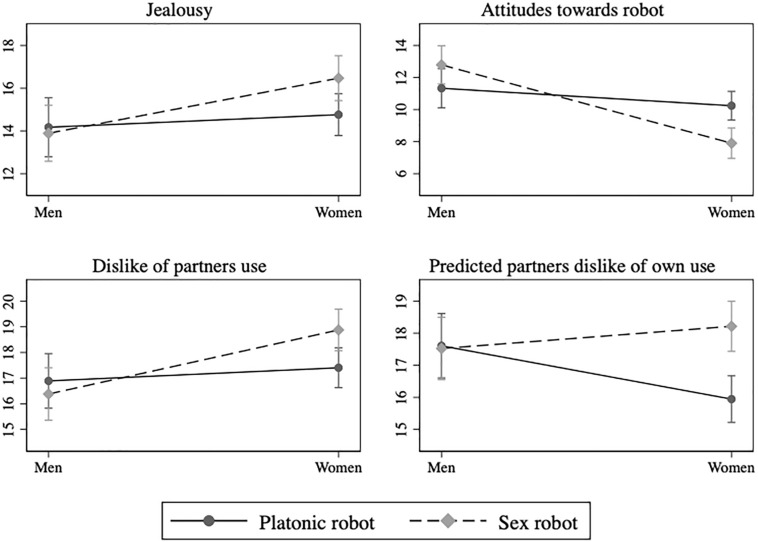
Interaction effects of gender and experimental condition on dependent variables.

Hypothesis 2 stated that male participants would be more positive toward robots if they had read a description of a sex robot, while female participants would be more positive toward robots if they had read a description of a platonic love robot. The results showed a significant negative interaction between gender and experimental condition confirming this hypothesis [*B* = −3.79, *p* < 0.01]. As seen in Figure 1, the interaction on attitudes between type of robot and gender was due mostly to the female participants disliking the sex robot, compared to the platonic love robot [*F*(1, 257) = 12.66, *p* < 0.01]. Males were more positive toward sex robots than platonic love robots, but not to a statistically significant degree. All in all the results suggest that males and females have very similar attitudes toward platonic love robots, but differ substantially in their attitudes toward sex robots, in that males are somewhat positive and females very negative to them.

Hypothesis 3 stated that males will expect to fell more jealous if their partner got a sex robot, while females would expect to feel more jealous if their partner got a platonic love robot. The results failed to provide support for this hypothesis. As mentioned, males expected to feel less jealous than females, regardless of type of robot their partner acquired. However, contrary to our expectations, the females expected to feel significantly more jealous if their partner acquired a sex robot, compared to females who envisioned that their partner acquired a platonic love robot [*F*(1, 257) = 5.57, *p* < 0.05].

Hypothesis 4 stated that that females would dislike the thought of their partner having a platonic love robot more, while males would dislike their partner having a sex robot. The results from the interaction model confirmed this hypothesis [*B* = 1.98, *p* < 0.05]. As seen in Figure 1, the small positive interaction effect is primarily due to the difference in predicted dislike at the thought of their partners use of the sex robot compared with a platonic love robot [*F*(1, 257) = 6.80, *p* < 0.01]. Male participants reported statistically similar levels of predicted dislike, regardless of what type of robot they had read about.

The fifth and final hypothesis suggested that males would expect their partner to dislike it more if he acquired a platonic love robot, while females would demonstrate the opposite pattern. This expectation was founded on the idea that the participants would project their own feelings onto their partners. The results provided support for such a projection account and showed a significant positive interaction effect [*B* = 2.35, *p* < 0.01]. Also seen in Figure 1, female participants expected their partners to dislike her having a sex robot, but be more comfortable with her having a platonic love robot [*F*(1, 257) = 17.81, *p* < 0.01]. By contrast, male participants expected their partners to be equally negative to him having either kind of robot.

## Discussion

The results of the analysis confirms previous findings that males are more positive toward the advent of robots than females (Scheutz and Arnold, 2016). Females who had read about the sex robot reported particularly elevated levels of jealousy, less favorable attitudes, more dislike and more predicted partner’s dislike. This pattern was not found in the male sample, whose feelings were largely unaffected by the type of robot they were made to envision.

One possible explanation for the gender difference could be a combination of differences in how males and females frame the concept of human-robot sexual relations, as well as different attitudes toward masturbation and the use of artificial stimulants for masturbatory purposes. Past research has indicated that males masturbate more, have more permissible attitudes toward masturbation, use more pornography, and have more permissive views of pornography consumption (Baumeister et al., 2001; Petersen and Hyde, 2010; Regnerus et al., 2016; Maas et al., 2018). If the males in the present study framed the prospect of having sex with robots as allegorically to masturbation with pornography, while the females considered the act more allegorical to cheating, one would expect the present results to emerge. While we did not include measures of how the participants view sex with robots, past research has suggested that males tend to think of sex with robots as a form of masturbation, not sex (Scheutz and Arnold, 2016). The overall gender difference in attitudes may also be partly due to men expressing their positive views more readily, while women may explicitly or implicitly not want positive attitudes toward robots. Future research should explore the moral and relational framing of human-robot sex in depth, including potential gender differences therein.

A different explanation for the observed results is that sex dolls and sex robots to this day primarily have been marketed toward men (Danaher and McArthur, 2017). This can explain why this idea evokes stronger negative feelings among females. In addition, the men and women might react differently to the lack of strong social cues in the sex-robot. According to the Persuasive robot’s acceptance model (Shazwani binti Ghazali, 2019), social cues and a lack of social cues predict attitudes toward robots. Women may view the sex robot in a more negative way both because they do not observe social cues and do not have an immediate sexual response. The observed gender differences may also be partly due to men and women finding it difficult to visualize forming a romantic bond with a non-human entity. Interestingly, studies have revealed that people seem to assume a more mutual relationship even with completely non-social service robots like vacuum cleaners (Forlizzi and DiSalvo, 2006; Sung et al., 2007). Such findings suggest that people get deeply engaged with robots even without humanoid qualities. However, the current study suggest that this effect may only be present in true interaction, not when anticipating future interaction, as our results indicate relatively small effects.

Findings from evolutionary psychology has generally indicated that females experience more jealousy at the thought of their partner having a romantic bond with another person, while males experience more jealousy at the thought of their female partner having a sexual relationship with another man (Buss et al., 1992, 1996, 1999). This finding has been explained by the different evolutionary imperatives faced by males and females. In a pre-industrial state, males had to compete for reproductive resources, and could know for certain whether the offspring they provide valuable resources to were actually related to them. Males have therefore developed their feelings of jealousy as an adaptive strategy to motivate behaviors that reduce paternity uncertainty and loss of access to reproductive resources. Their jealousy is thus especially attuned to the threat of sexual encounters. Females, on the other hand, faced certainty in their rightful motherhood, but face the risk of their partner abandoning her and their common offspring, which severely compromises the odds of survival. Their jealousy is thus geared less toward purely sexual escapades without any other forms of attachment, and more concerned with emotional bonds that may distract paternal investment in partner and offspring. This adaptation account has been proposed as a the explanation for the observed gender differences across cultures (Buss and Haselton, 2005). One problem facing this account is that it can be difficult for participants to envision their partner in a purely emotional or purely sexual relationship with someone, without envisioning that the relationship can change and evolve over time. A purely romantic attraction can evolve into a sexual one, and vice versa. In this study, however, we offer a more “clean” manipulation of this variable, in that the robots we described were either purely sexual or purely non-sexual. The sex robot was explicitly described as unable to engage in anything more than a sexual relationship, while the platonic love robot was explicitly described as disembodied and unable to satisfy physical sexual urges. Our findings therefore shed new light on how males and females feel about different kinds of infidelity in a setting where sex cannot lead to love and love cannot lead to sex.

Our results further show that males and females varied in how they expected to feel if their partner acquired and used a sex robot or platonic love robot. However, the results demonstrate that both males and females fail to predict how their partner would feel if they themselves got a robot. Males, who report feeling at ease with the thought of their partner having a robot, erroneously expect that their partners will extend the same relaxed attitude toward them. Females on the other hand, who are negative to the prospect of their male partners having a sex robot, and neutral to them having a platonic love robot, erroneously expect their partners to react negatively to them having a sex robot and positively to them having a platonic love robot. These results are in line with a projection account, which suggests that people tend to expect their partners to feel as they would have, especially in emotionally charged situations (Newman et al., 1997; Kawada et al., 2004; Maner et al., 2005).

### Limitations

There are two notable limitations to the present study. The first is the recruitment procedure and sample. Participants were recruited primarily via social media (Facebook) and accessible e-mail lists to workplaces. Therefore, our sample is likely to be influenced by a self-selection bias, whereby those who thought human-robotic interaction more interesting presumably were more likely to participate in the study. The sample of participants consisted of a majority of students, and was somewhat restricted in age variation, which limits the generalizability of the findings. In addition, the results cannot be directly generalized to homosexual populations as the sample was almost exclusively heterosexual. The second limitation is the use of novel non-validated measurements. There are few validated measurements of reactions to robots, and to the best of our knowledge, none that capture sentiments regarding sex and love robots. The *Negative Attitudes toward Robots Scale* (NARS) (Nomura et al., 2006b) is too general for the purposes of our study. In order to gain thorough understanding of how people feel about different types of robots designed for physical and emotional intimacy, improved measurement scales need to be designed and validated.

## Conclusion

Physical and emotional intimacy with robots may become more commonplace over the next decades, as technology improves at a rapid rate. The results of the current study show that women have less positive views of robots, and especially of sex robots, compared to men. The results further suggests that people project their own feelings about robots onto their partner, erroneously expecting their partner to react as they would to the thought of ones’ partner having a robot.

## Data Availability Statement

The datasets generated for this study are available on request to the corresponding author.

## Ethics Statement

The studies involving human participants were reviewed and approved by the Research Ethics Committee at BI Norwegian Business School, Campus Bergen. The participants provided their written informed consent to participate in this study.

## Author Contributions

All authors listed have made a substantial, direct and intellectual contribution to the work, and approved it for publication.

## Conflict of Interest

The authors declare that the research was conducted in the absence of any commercial or financial relationships that could be construed as a potential conflict of interest.

## References

[B1] Baumeister R. F. Catanese K. R. Vohs K. D. (2001). Is there a gender difference in strength of sex drive? theoretical views, conceptual distinctions, and a review of relevant evidence. *Personal. Soc Psychol. Rev.* 5 242–273. 10.1207/S15327957PSPR0503_5

[B2] Beutel M. E. Klein E. M. Brähler E. Reiner I. Jünger C. Michal M. (2017). Loneliness in the general population: prevalence, determinants and relations to mental health. *BMC Psychiatry* 17:97. 10.1186/s12888-017-1262-x 28320380 PMC5359916

[B3] Buss D. M. Haselton M. (2005). The evolution of jealousy. *Trends Cogn. Sci.* 9 506–506. 16199197 10.1016/j.tics.2005.09.006

[B4] Buss D. M. Larsen R. J. Westen D. (1996). Sex differences in jealousy: not gone, not forgotten, and not explained by alternative hypotheses. *Psychol. Sci.* 7 373–375. 10.1111/j.1467-9280.1996.tb00392.x

[B5] Buss D. M. Larsen R. J. Westen D. Semmelroth J. (1992). sex differences in jealousy: evolution, physiology, and psychology. *Psychol. Sci.* 3 251–256. 10.1111/j.1467-9280.1992.tb00038.x

[B6] Buss D. M. Shackelford T. K. Kirkpatrick L. A. Choe J. C. Lim H. K. Hasegawa M. (1999). Jealousy and the nature of beliefs about infidelity: tests of competing hypotheses about sex differences in the United States. Korea, and Japan. *Pers. Relationsh.* 6 125–150. 10.1111/j.1475-6811.1999.tb00215.x

[B7] Coyle C. E. Dugan E. (2012). Social isolation, loneliness and health among older adults. *J. Aging Health* 24 1346–1363. 10.1177/0898264312460275 23006425

[B8] Croson R. Gneezy U. (2009). Gender differences in preferences. *J. Econ. Literature* 47 448–474. 10.1257/jel.47.2.448

[B9] Danaher J. McArthur N. (2017). *Robot Sex: Social and Ethical Implications.* Cambridge, MA: MIT Press.

[B10] Dautenhahn K. Bond A. H. Canamero L. Edmonds B. (2006). *Socially Intelligent Agents: Creating Relationships with Computers and Robots.* Dordrecht: Kluwer Academic Publishers.

[B11] de Graaf M. M. A. Ben Allouch S. (2013). Exploring influencing variables for the acceptance of social robots. *Rob. Auton. Syst.* 61 1476–1486. 10.1016/j.robot.2013.07.007

[B12] Del Giudice M. (2011). Sex differences in romantic attachment: a meta-analysis. *Personal. Soc. Psychol. Bull.* 37 193–214. 10.1177/0146167210392789 21239594

[B13] Dindia K. Allen M. (1992). Sex differences in self-disclosure: a meta-analysis. *Psychol. Bull.* 112 106. 10.1037/0033-2909.112.1.106 1388280

[B14] Douglas S. P. Craig C. S. (2007). Collaborative and Iterative translation: an alternative approach to back translation. *Int. Market.* 15 30–43. 10.1509/jimk.15.1.030

[B15] Druin A. Hendler J. A. Hendler J. (2000). *Robots for Kids: Exploring New Technologies for Learning.* Los Altos, CA: Morgan Kaufmann.

[B16] Eagly A. H. Johnson B. T. (1990). Gender and leadership style: a meta-analysis. *Psychol. Bull.* 108:233. 10.1037/0033-2909.108.2.233 12848221

[B17] Feingold A. (1994). Gender differences in personality: A meta-analysis. *Psychol. Bull.* 116:429. 10.1037/0033-2909.116.3.429 7809307

[B18] Fischer C. S. (1988). Gender and the residential telephone, 1890–1940: Technologies of sociability. *Sociol. Forum* 3 211–233. 10.1007/BF01115291

[B19] Flandorfer P. (2012). Population ageing and socially assistive robots for elderly persons: the importance of sociodemographic factors for user acceptance. *Int. J. Popul. Res.* 2012 1–13. 10.1155/2012/829835

[B20] Forlizzi J. DiSalvo C. (2006). “Service robots in the domestic environment: a study of the roomba vacuum in the home,” in *Proceedings of the 1st ACM SIGCHI/SIGART Conference on Human-Robot Interaction*, (New York, NY: ACM), 258–265.

[B21] Frank L. Nyholm S. (2017). Robot sex and consent: is consent to sex between a robot and a human conceivable, possible, and desirable? *Artif. Intell. Law* 25 305–323. 10.1007/s10506-017-9212-y

[B22] Holt J. L. DeVore C. J. (2005). Culture, gender, organizational role, and styles of conflict resolution: A meta-analysis. *Int. J. Int. Relat.* 29 165–196. 10.1016/j.ijintrel.2005.06.002

[B23] Holt-Lunstad J. Smith T. B. Baker M. Harris T. Stephenson D. (2015). Loneliness and social isolation as risk factors for mortality: a meta-analytic review. *Perspect. Psychol. Sci.* 10 227–237. 10.1177/1745691614568352 25910392

[B24] Ishiguro H. Ono T. Imai M. Kanda T. (2003). “Development of an interactive humanoid robot “Robovie”: an interdisciplinary approach,” in *Robotics Research*, eds Jarvis R. A. Zelinsky A. (Berlin: Springer), 179–191. 10.1007/3-540-36460-9_12

[B25] Kawada C. L. Oettingen G. Gollwitzer P. M. Bargh J. A. (2004). The projection of implicit and explicit goals. *J. Personal. Soc. Psychol.* 86: 545. 10.1037/0022-3514.86.4.545 15053705

[B26] Levy D. (2007). *Love and Sex with Robots: The Evolution of Human-Robot Relationships.* New York, NY: Harper Collins.

[B28] Maas M. K. Vasilenko S. A. Willoughby B. J. (2018). A dyadic approach to pornography use and relationship satisfaction among heterosexual couples: the role of pornography acceptance and anxious attachment. *J. Sex Res.* 55 772–782. 10.1080/00224499.2018.1440281 29578817 PMC6155976

[B29] Maner J. K. Kenrick D. T. Becker D. V. Robertson T. E. Hofer B. Neuberg S. L. (2005). Functional projection: How fundamental social motives can bias interpersonal perception. *J. f Personal. Soc. Psychol.* 88 63–78. 10.1037/0022-3514.88.1.63 15631575

[B30] Nam S. K. Chu H. J. Lee M. K. Lee J. H. Kim N. Lee S. M. (2010). A meta-analysis of gender differences in attitudes toward seeking professional psychological help. *J. Am. Coll. Health* 59 110–116. 10.1080/07448481.2010.483714 20864437

[B31] Newman L. S. Duff K. J. Baumeister R. F. (1997). A new look at defensive projection: thought suppression, accessibility, and biased person perception. *J. Personal. Soc. Psychol.* 72:980. 10.1037/0022-3514.72.5.980 9150580

[B32] Niedenthal P. M. Halberstadt J. B. Margolin J. Innes-Ker A. H. (2000). Emotional state and the detection of change in facial expression of emotion. *Eur. J. Soc. Psychol.* 30 211–222. 10.1002/(sici)1099-0992(200003/04)30:2<211::aid-ejsp988>3.0.co;2-3

[B33] Nolen-Hoeksema S. (2012). Emotion regulation and psychopathology: the role of gender. *Annu. Rev. Clin. Psychol.* 8 161–187. 10.1146/annurev-clinpsy-032511-143109 22035243

[B34] Nomura T. Kanda T. (2003). “On proposing the concept of robot anxiety and considering measurement of it,” in *Proceedings of the twelveth IEEE International Workshop on Robot and Human Interactive Communication*, Millbrae, CA, 373–378. 10.1109/ROMAN.2003.1251874

[B35] Nomura T. Kanda T. Suzuki T. (2006a). Experimental investigation into influence of negative attitudes toward robots on human–robot interaction. *Ai Soc.* 20 138–150. 10.1007/s00146-005-0012-7

[B36] Nomura T. Kanda T. Suzuki T. Kato K. (2006b). “Exploratory investigation into influence of negative attitudes toward robots on human-robot interaction,” in *Mobile Robots: Towards New Applications*, (London: IntechOpen.).

[B37] O’Brien K. E. Biga A. Kessler S. R. Allen T. D. (2010). A meta-analytic investigation of gender differences in mentoring. *J. Manag.* 36 537–554. 10.1177/0149206308318619

[B38] Petersen J. L. Hyde J. S. (2010). A meta-analytic review of research on gender differences in sexuality, 1993–2007. *Psychol. Bull.* 136 21–38. 10.1037/a0017504 20063924

[B39] Regnerus M. Gordon D. Price J. (2016). Documenting pornography use in America: a comparative analysis of methodological approaches. *J. Sex Res.* 53 873–881. 10.1080/00224499.2015.1096886 26683998

[B40] Richardson K. (2016). Sex robot matters: slavery, the prostituted, and the rights of machines. *IEEE Technol. Soc. Mag.* 35 46–53. 10.1109/MTS.2016.2554421

[B41] Sanders T. (2013). *Paying for Pleasure: Men Who Buy Sex*. New York, NY: Routledge.

[B42] Schermerhorn P. Scheutz M. Crowell C. R. (2008). “Robot social presence and gender: do females view robots differently than males?,” in *Proceedings of the 3rd ACM/IEEE International Conference on Human Robot Interaction*, (New York, NY: ACM), 263–270.

[B43] Scheutz M. Arnold T. (2016). “Are we ready for sex robots?,” in *The Eleventh ACM/IEEE International Conference on Human Robot Interaction*, Christchurch, 351–358.

[B44] Shazwani binti Ghazali A. (2019). *Designing Social Cues for Effective Persuasive Robots.* Eindhoven: Technische Universiteit Eindhoven.

[B45] Shoham Y. Perrault R. Brynjolfsson E. Clark J. Manyika J. Niebles J. C. (2018). *The AI Index 2018 Annual Report.* Stanford, CA: Stanford University.

[B46] Sullins J. P. (2012). Robots, love, and sex: the ethics of building a love machine. *IEEE Trans. Affect. Comput.* 3 398–409. 10.1109/T-AFFC.2012.31

[B47] Sung J.-Y. Guo L. Grinter R. E. Christensen H. I. (2007). “My roomba is rambo”: intimate home appliances,” in *International Conference on Ubiquitous Computing*, (Berlin: Springer), 145–162. 10.1007/978-3-540-74853-3_9

[B48] Tamres L. K. Janicki D. Helgeson V. S. (2002). Sex differences in coping behavior: a meta-analytic review and an examination of relative coping. *Personal. Soc. Psychol. Rev.* 6 2–30. 10.1207/S15327957PSPR0601_1

[B49] Victor C. R. Yang K. (2012). The prevalence of loneliness among adults: a case study of the United Kingdom. *J. Psychol.* 146 85–104. 10.1080/00223980.2011.613875 22303614

[B50] Walters A. E. Stuhlmacher A. F. Meyer L. L. (1998). Gender and negotiator competitiveness: a meta-analysis. *Organ. Behav. Hum. Decis. Process.* 76 1–29. 10.1006/obhd.1998.2797 9756737

[B51] Wright P. H. Scanlon M. B. (1991). Gender role orientations and friendship: some attenuation, but gender differences abound. *Sex Roles* 24 551–566. 10.1007/BF00288413

[B52] Yarnell L. M. Stafford R. E. Neff K. D. Reilly E. D. Knox M. C. Mullarkey M. (2015). Meta-analysis of gender differences in self-compassion. *Self and Identity* 14 499–520. 10.1080/15298868.2015.1029966

[B53] Yeoman I. Mars M. (2012). Robots, men and sex tourism. *Futures* 44 365–371. 10.1016/j.futures.2011.11.004

[B54] Young J. E. Hawkins R. Sharlin E. Igarashi T. (2008). Toward acceptable domestic robots: applying insights from social psychology. *Int. J. Soc. Rob* 1:95 10.1007/s12369-008-0006-y

