# Supplementary material for: Friends, Lovers or Nothing: Men and Women Differ in Their Perceptions of Sex Robots and Platonic Love Robots
Source: Front Psychol. 2020 Mar 13;11:355. doi: 10.3389/fpsyg.2020.00355 (PMC7083111; doi:10.3389/fpsyg.2020.00355)
Supplement: Supplementary file 1 [file Data_Sheet_1.PDF]

Appendix

Platonic love robot:

Imagine the year 2035. The world has seen great advances in artificial intelligence and robotics. One of the advances has led to the development of highly realistic love robots, both in male and female form. The robots able to talk to their owners in a way that feels very human-like and realistic. They have a natural sounding voice, perfect memory, and are experienced as highly and genuinely empathetic. The robots understand humor and emotions. The artificial intelligence the robots are equipped with enables them to get to know their owner through experience. They adapt their communication accordingly, so that the emotional needs of the owner are tended to. User surveys show that the owners of this kind of love robot are extremely satisfied Both male and female users report experiencing a close and deep relationship with their robot, and that they talk with it every day. Many users say that whenever something good or bad happens in their life, their robot is the first one they want to share the news with. Many also say that the robot is the entity with which they share most personal secrets.

Even though the love robots are equipped with a highly sophisticated artificial intelligence, there are some limitations to them. The robots have no physical body, it only exist in a small microphone and speaker. This means that the robot is unable to have a physical or sexual relationship with its owner. It can form a meaningful romantic and friendly relation to a human, but it cannot satisfy the owner in a sexual manner.

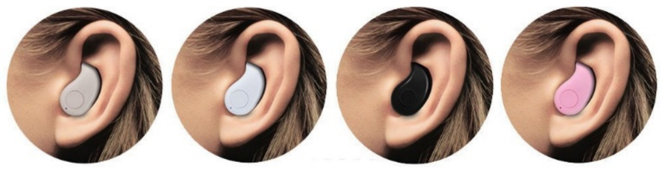

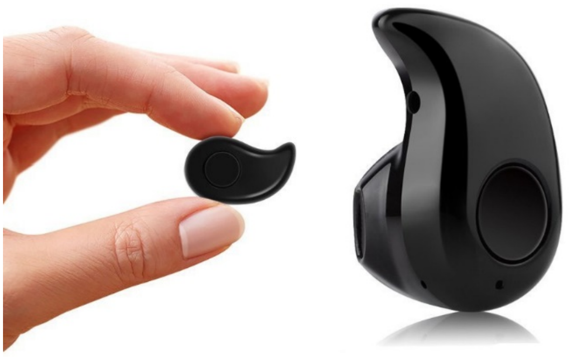

Sex robot:

Imagine the year 2035. The world has seen great advances in artificial intelligence and robotics. One of the advances has led to the development of highly realistic sex robots, both in male and female form. The robots looks and feels just like humans. They have highly realistic skin and hair, and feel and smell just like real humans. The bodies have a highly natural patterns of movement, and well defined proportions. The artificial intelligence the robots are equipped with enables them to learn their owner's sexual preferences through experience, and adapt their sexual behavior patterns accordingly, so that the sexual needs of the owner are tended to. User surveys show that the owners of this kind of sex robot are extremely satisfied. Both male and female users report having very pleasant experiences with their robot, that they use it several times per week, and that they are able to achieve orgasm with the robot.

Even though the sex robots are equipped with a highly sophisticated artificial intelligence, there are some limitations to them. The robots can only have a sexual relationship with their owner. Attempts of non-sexual interactions will either be misunderstood, ignored or interpreted in a sexual way by the robot. This means that the robots are unable to satisfy any other needs, besides sexual needs. The robots cannot form a meaningful romantic or friendly relation with a human.

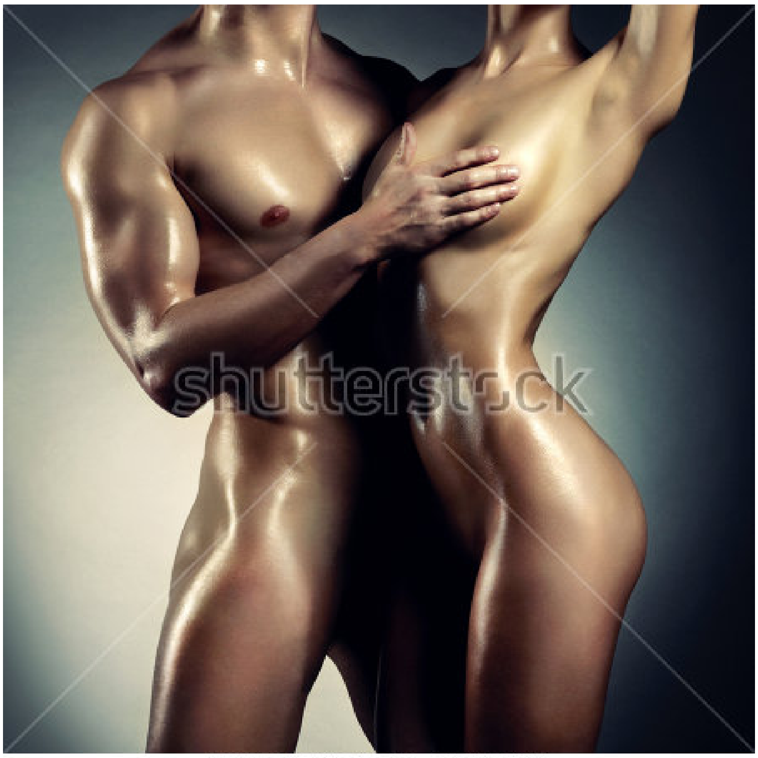

www.shutterstock.com · 148724903
